# Antimicrobial Activity and Characterization of a Validated Copper-Complexed Polymer Tape for Surface Disinfectant Applications

**DOI:** 10.3390/antibiotics14121262

**Published:** 2025-12-14

**Authors:** Andreanne G. Vasconcelos, William D. Amorim, Bruno S. Sá, Luan B. V. Costa, Gustavo S. de Araujo, Helder Andrey R. Gomes, Jorge Antônio Chamon Júnior, Amabel F. Correia, Íris Cabral, Thales R. Machado, Dayse Maria C. de Mendonça, Ingrid Gracielle M. da Silva, Joaquim L. Júnior, Elivaldo R. de Santana, Yvonne Mascarenhas, Sônia N. Báo, Valtencir Zucolotto, Peter Eaton, Ciro M. Gomes, José Roberto de S. A. Leite

**Affiliations:** 1 People&Science Pesquisa Desenvolvimento e Inovação Ltda, Centro de Apoio ao Desenvolvimento Tecnológico (CDT), University of Brasília (UnB), Brasília 70910-900, DF, Brazil; 2 Research Center in Applied Morphology and Immunology (NuPMIA), Faculty of Medicine, University of Brasília (UnB), Brasília 70910-900, DF, Brazil; 3 Centro Universitário do Distrito Federal (UDF), Brasília 70390-045, DF, Brazil; 4 The Bridge, University of Lincoln, Joseph Ruston Building, Lincoln LN6 7EL, UK; peaton@lincoln.ac.uk; 5 Programa de Pós-Graduação em Patologia Molecular, Faculty of Medicine, University of Brasília (UnB), Brasília 70910-900, DF, Brazil; 6 Laboratório Central de Saúde Pública (LACEN-DF), Brasília 70830-010, DF, Brazil; 7 Nanomedicine and Nanotoxicology Group, São Carlos Institute of Physics, University of São Paulo (USP), São Carlos 13566-590, SP, Brazilzuco@ifsc.usp.br (V.Z.); 8 Gerência de Atenção à Saúde, Unidade de Clínica Médica, Hospital Universitário de Brasília (HUB), University of Brasília (UnB), Brasília 72830-200, DF, Brazil; 9 Laboratório de Microscopia e Microanálise, Instituto de Ciências Biológicas, University of Brasília (UnB), Brasília 70910-900, DF, Brazilsnbao@unb.br (S.N.B.); 10 Programa de Epidemiologia e Vigilância em Saúde (PEPIVS), Fundação Oswaldo Cruz (Fiocruz), Brasília 70910-900, DF, Brazil; 11 Laboratório de Simulação da Faculdade de Medicina, University of Brasília (UnB), Brasília 70910-900, DF, Brazil; 12 São Carlos Institute of Physics, University of São Paulo (USP), São Carlos 13566-590, SP, Brazil; yvonne@ifsc.usp.br; 13 Unidade de Dermatologia, Hospital Universitário de Brasília (HUB), University of Brasília (UnB), Brasília 72830-200, DF, Brazil; cirogomes@unb.br

**Keywords:** antimicrobial coatings, copper-complexed polymers, multidrug-resistant bacteria, healthcare-associated infections, surface disinfection, infection control

## Abstract

**Background**: Surface contamination in healthcare environments plays a key role in the persistence and transmission of microorganisms. Long-lasting antimicrobial coatings based on copper–polymer complexes offer a promising passive strategy to minimize environmental contamination and healthcare-associated infections. **Methods**: This study evaluated a copper-alloy polymeric tape through physicochemical, *in vitro*, and hospital-based assessments. Structural analyses (XRD, Raman, SEM, EDX) were used to characterize the material, while antimicrobial efficacy was determined against Gram-positive and Gram-negative bacteria following ISO 22196:2011. A randomized 19-week clinical study was conducted in the Emergency and Urgent Unit of the University Hospital of Brasília to quantify microbial loads on high-touch surfaces covered with the copper-alloy tape or a non-antimicrobial control. **Results**: Structural characterization techniques validated the integrity and heterogeneous distribution of copper within the polymeric matrix. All tested bacterial strains exhibited complete growth inhibition on the copper-alloy tape, with final counts consistently below the detection threshold (<1.00 log10 CFU/mL). Human keratinocytes analyzed by SEM showed preserved morphology. In hospital conditions, treated surfaces maintained significantly lower microbial loads than controls over 19 weeks. The number of yeast-positive samples was small compared to the total number of samples collected during the study, but *Candida parapsilosis* was the most frequently identified species. **Conclusions**: These findings support its use as a sustainable intervention to reduce environmental contamination and infection risks in healthcare settings.

## 1. Introduction

Infectious diseases are significant causes of morbidity and mortality worldwide. It is estimated that infections caused by bacteria resistant to clinically used antimicrobial drugs contribute to more than 4 million deaths annually [1]. Additionally, fungal infections are a growing concern, particularly for immunocompromised individuals, patients in Intensive Care Units (ICUs), those undergoing invasive procedures, or those receiving broad-spectrum antibiotic therapy [2]. Moreover, the spread of antifungal resistance has significant implications for human health [3].

Increased global connectivity, along with demographic and climate changes, enables pathogens to reach new environments rapidly, spreading infectious diseases. The introduction of new pathogens can have widespread impacts on populations and ecosystems, as evidenced recently by the COVID-19 pandemic. However, ensuring consistent hygiene practices, including appropriate procedures and frequencies, remains a challenge, as it requires both awareness and behavioral change. In this context, the development of surface coatings with antimicrobial properties has garnered increasing interest, as they offer a promising strategy for large-scale control of the spread of bacterial, fungal, and viral pathogens [4].

Copper has been extensively studied for its antimicrobial properties against a wide range of pathogens, including bacteria, yeast and viruses [5]. The antimicrobial action of copper is attributed to its ability to disrupt microbial cell membranes and induce oxidative stress through the generation of reactive oxygen species (ROS), leading to protein, lipid, and DNA damage [5,6]. Additionally, copper ions can interfere with essential cellular processes by binding to enzymes and nucleic acids, inhibiting microbial respiration and replication [5,6].

Research has shown that metallic copper surfaces can effectively reduce bacterial contamination in hospital settings, particularly for multidrug-resistant strains like Methicillin-resistant *Staphylococcus aureus* (MRSA), Carbapenem-resistant Enterobacteriaceae (CRE) and different types of *Candida* [6,7]. The broad-spectrum efficacy of copper, combined with its low risk of resistance development, makes it a promising candidate for antimicrobial coatings and medical applications. Nevertheless, the use of solid copper is often impractical due to high cost as well as inferior mechanical properties compared to competing materials such as reinformed plastics or other metals [8]. For this reason, copper containing materials are of interest.

Beyond healthcare settings, contaminated high-touch surfaces are well-documented sources of pathogen transmission in public transportation, food processing industries, and community environments [9,10,11]. Traditional cleaning and disinfection protocols are effective but highly dependent on proper implementation and frequency, leaving gaps for microbial survival and cross-contamination [12]. Thus, integrating long-lasting antimicrobial coatings directly onto frequently touched materials is emerging as a sustainable and passive intervention to mitigate the spread of multidrug-resistant organisms [13]. Copper-based coatings, especially when complexed with polymeric matrices, offer enhanced durability, better adhesion to diverse substrates, and optimized antimicrobial efficacy, making them attractive candidates for real-world application [14,15].

In this study, we present the physicochemical characterization and the antimicrobial performance—both *in vitro* and in a hospital environment—of a validated copper-complexed polymer coating designed for infection prevention. To our knowledge, this is the first independent validation of this type of copper-based antimicrobial adhesive material under real hospital conditions in Brazil, considering the diverse environmental microbiota and healthcare challenges characteristic of tropical regions. Additionally, because the performance of copper-based antimicrobial surfaces can be modulated by environmental humidity, validating this material under the high-moisture conditions typical of Brazilian healthcare settings provides essential evidence not yet addressed in prior evaluations. The findings highlight the potential of this copper-alloy tape as a sustainable solution for reducing healthcare-associated infections and environmental contamination.

## 2. Results and Discussion

The development of antimicrobial surface coatings requires not only the demonstration of their microbicidal capacity but also a clear understanding of how their physicochemical characteristics influence biological performance. In this study, we combined structural analyses and microbiological assays to explore the antimicrobial potential of a copper-complexed polymer tape designed for application on high-touch surfaces. By integrating quantitative assays with imaging approaches, the results provide complementary evidence of the coating’s efficacy, its modes of action against bacterial cells, and its potential compatibility with mammalian cells. This multifaceted evaluation allows for a critical assessment of that copper-alloy tape in the context of existing copper-based surface technologies and highlights its possible applications in healthcare and beyond.

### 2.1. Physicochemical Characteristics

**Figure 1:**
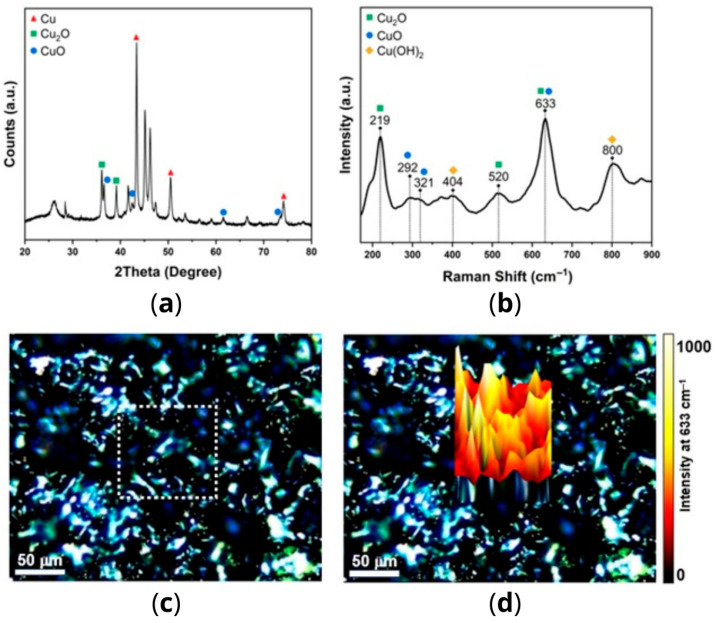
(**a**) XRD pattern of the copper-alloy tape; (**b**) Raman spectrum of the copper-alloy tape; (**c**) optical micrograph of the tape surface with the region designated for Raman mapping outlined by a dotted square; (**d**) 3D Raman intensity mapping obtained by monitoring the Cu_2_O/CuO band at 633 cm^−1^.

The diffraction pattern obtained by X-ray diffraction (XRD) of the sample is shown in Figure 1a. The intense and defined peaks indicate the presence of well-crystallized phases. The peaks at 43.3°, 50.6°, and 74.2° are associated with the families of planes (111), (200), and (220) of the cubic phase from metallic Cu (PDF#4-836), with this phase being predominantly present. Meanwhile, the peaks at 36.6°, 42.3°, 61.5°, and 73.6° are attributed to the families of planes (111), (200), (220), and (311) of the cubic phase of Cu_2_O (PDF#75-1531). Lastly, the peaks at 36.0° and 39.1° are attributed to the families of planes (−111) and (111) of the monoclinic phase of CuO (PDF#74-1021). The other peaks can be attributed to the polymeric matrix.

The copper-alloy tape was analyzed using Raman spectroscopy, and the corresponding single spectrum is illustrated in Figure 1b. The identified bands are associated with various copper oxides, including Cu_2_O (219 and 520 cm^−1^), CuO (292 and 321 cm^−1^), and Cu(OH)_2_ (404 and 800 cm^−1^) [16,17]. Additionally, the asymmetric band at 633 cm^−1^ is a result of overlapping bands of Cu_2_O and CuO [16]. These results corroborate the findings obtained by XRD in identifying the copper states present in the sample, except for the metallic Cu, which is not Raman-active. To reveal the spatial distribution of these copper oxides on copper-alloy tape, Raman mapping was employed. Figure 1c displays an optical micrograph of the analyzed area marked by the white dotted square, while Figure 1d presents the 3D reconstruction achieved by monitoring the intensity of the 633 cm^−1^ band. The results indicate an uneven distribution of copper oxides across the sample surface, with certain regions exhibiting higher concentrations, evident from the yellowish-white peaks in the color map [16,17].

**Figure 2:**
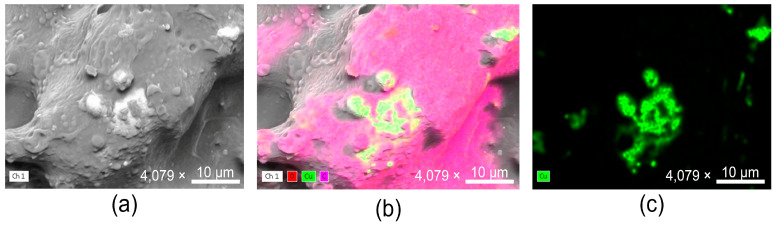
(**a**) SEM micrograph of the copper-alloy tape surface showing a heterogeneous topography characterized by roughness and irregular projections; (**b**,**c**) EDX elemental maps confirming the presence and distribution of copper (Cu, green signal), oxygen (O, red signal), and carbon (C, magenta signal) across the analyzed area. Images are representative of three independent surface analyses performed under identical conditions.

The morphology and elemental composition of copper-alloy tape were investigated using Scanning Electron Microscopy (SEM) coupled with Energy-Dispersive X-Ray Spectroscopy (EDX). The SEM image (Figure 2a) revealed a heterogeneous surface characterized by irregularities and roughness, features typically associated with polymer–metal composite coatings. Such micro- and nanoscale heterogeneity is relevant, as it increases the available contact area and may influence the interaction of the coating with microbial cells and environmental factors.

Elemental mapping by EDX (Figure 2b,c) confirmed the presence and distribution of copper across the analyzed surface. The copper signal appeared well dispersed, with regions of higher intensity corresponding to localized clusters of deposition. Oxygen and carbon were also detected, consistent with the polymeric matrix that anchors the metallic phase. The coexistence of these elements corroborates the structural analyses obtained by XRD and Raman spectroscopy, which demonstrated the presence of copper oxides integrated within the polymer network.

Together, the findings from RAMAN spectroscopy, along with SEM and EDX analyses, provide evidence that the tape exhibits a rough surface and widespread distribution of copper within the polymeric matrix, with localized regions of higher deposition intensity. These characteristics are crucial for ensuring sustained antimicrobial activity, as they support the continuous exposure of copper at the interface and suggest durability of the coating under real-use conditions [18].

### 2.2. Antimicrobial Performance and Microscopic Characterization of Biological Interactions

The antimicrobial efficacy of the copper-alloy tape is presented in Table 1. After 24 h of exposure, all tested bacterial strains exhibited complete growth inhibition on the copper-alloy tape, with final counts consistently below the detection threshold (<1.00 log10 CFU/mL). In contrast, control samples without the antimicrobial agent maintained high levels of viable bacteria, with mean values of 5.33 ± 0.61 log10 CFU/mL for *Escherichia coli*, 5.22 ± 0.54 log10 CFU/mL for *Pseudomonas aeruginosa*, and 3.59 ± 0.53 log10 CFU/mL for *Salmonella enterica* subsp. enterica serovar Choleraesuis. For *Staphylococcus aureus*, the control group showed relatively low counts (1.66 ± 0.72 log10 CFU/mL), which precluded the calculation of log reduction according to ISO 22196 criteria [19]; nonetheless, no viable cells were detected on the treated films, indicating a clear bactericidal effect.

Overall, copper-alloy tape achieved reductions exceeding 4 log10 (>99.99%) for *E. coli* and *P. aeruginosa* and 2.58 log10 (99.7%) for *S. enterica* after 24 h of contact. These values are consistent with the highest levels of antimicrobial performance reported for copper-based coatings [20]. Comparable findings have been described for copper-containing stainless steel, which achieve approximately 100% killing of *E. coli* and *S. aureus* after 48 h of contact with the surface [21].

Importantly, the observed activity against *S. enterica* expands the potential application of copper-alloy tape beyond healthcare environments to the food industry, where enteric pathogens remain a major concern. Compared with other copper-based surface technologies [14,18], the present results place copper-alloy tape among the most effective coatings reported to date, supporting its integration as a passive, durable intervention for infection prevention and control in both clinical and community settings.

**Figure 3:**
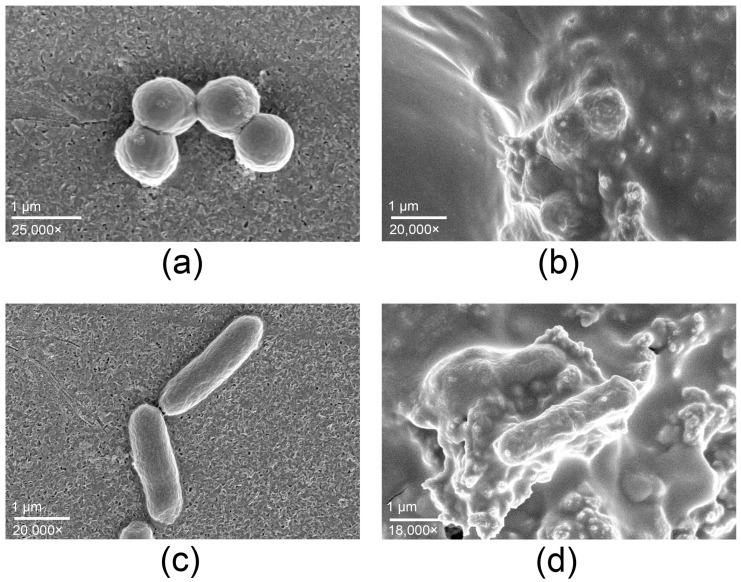
*Staphylococcus aureus* (**a**,**b**) and *Salmonella typhimurium* (**c**,**d**) observed by SEM. Control samples (**a**,**c**) show intact cell morphology, whereas cells exposed to the copper-alloy tape (**b**,**d**) exhibit pronounced structural alterations, including membrane rupture and accumulation of cellular debris, consistent with copper-mediated antimicrobial activity.

The SEM micrographs (Figure 3) provide complementary evidence supporting the antimicrobial activity observed. Untreated controls exhibited intact cell morphology, spherical *S. aureus* cells (Figure 3a) and rod-shaped *Salmonella typhimurium* (Figure 3c), both displaying smooth surfaces. In contrast, cells exposed to the copper-alloy tape showed pronounced structural alterations. *S. aureus* (Figure 3b) presented collapsed cell walls and accumulation of surface debris, whereas *S. typhimurium* (Figure 3d) displayed membrane rupture, deformation, and adhesion of lysed material.

The log10 reductions obtained under ISO 22196 conditions, together with the SEM evidence of cellular disruption, indicate that the copper-alloy tape exhibits high antimicrobial efficacy. The broad-spectrum efficacy of copper-alloy tape and the observations obtained by SEM agree with the known antimicrobial mechanisms of copper and copper oxides, which include membrane disruption, oxidative stress induction, and damage to proteins and nucleic acids [6]. The notable activity against *Salmonella* suggests that its application may extend beyond healthcare-associated infection control to environments where surface contamination is a concern, such as food handling or processing areas.

**Figure 4:**
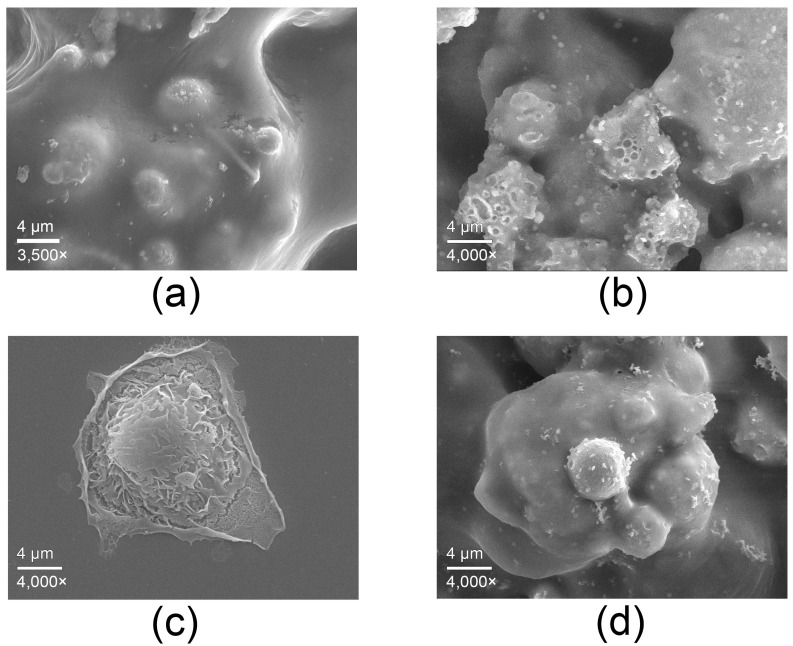
Micrographs obtained by SEM. (**a**) Surface of the copper-alloy tape included for reference; (**b**) surface of the copper-alloy tape after incubation with culture medium alone; (**c**) control HaCaT cells; (**d**) HaCaT cells after 6 h of contact with the copper-alloy tape.

SEM analysis (Figure 4) illustrates the interaction of human keratinocytes (HaCaT) with the surface of the copper-alloy tape. Figure 4a shows the surface of the copper-alloy tape as previously characterized, included here for reference and comparison with the images of cell interaction. Figure 4b shows the surface of the copper-alloy tape after incubation with culture medium alone, revealing a small crater appearance due to salt deposition from the medium.

Keratinocytes in contact with the copper-alloy tape for 6 h (Figure 4d) adhered to the surface and exhibited a slightly retracted and rounded morphology compared to the control cell (Figure 4c), but maintained intact membranes and surface structures, with no evidence of physical damage. This behavior indicates moderate morphological adaptation to the metal surface, rather than cytotoxic effects.

These findings suggest that the copper-alloy tape appears to support short-term contact with mammalian cells under controlled *in vitro* conditions, while maintaining its antimicrobial properties demonstrated in previous assays. Although not sufficient to establish biocompatibility, these results are consistent with the dual functionality expected for copper-based antimicrobial surfaces and support the potential applicability of this material in healthcare settings where both antimicrobial performance and material safety are relevant considerations.

### 2.3. Clinical Assessment in Hospital Environment

The evaluation of the antimicrobial activity of copper-alloy tape under real-world environmental conditions was conducted in the Emergency and Urgent Unit (EUU) of the University Hospital of Brasília (HUB) for 19 consecutive weeks. The locals selected—patient unit divider curtains (external surface), chair armrests (right and left), drawer handles of medication carts, sink faucets in the EUU, sink faucets in the bathrooms, and grab bars in the bathrooms (shower and toilet bowl)—represent critical reservoirs of persistent contamination in healthcare settings. In addition to analyzing individual surfaces, a global value was calculated, representing the average number of colony-forming units (CFUs) across all location categories, thus providing an integrated measure of antimicrobial performance.

**Figure 5:**
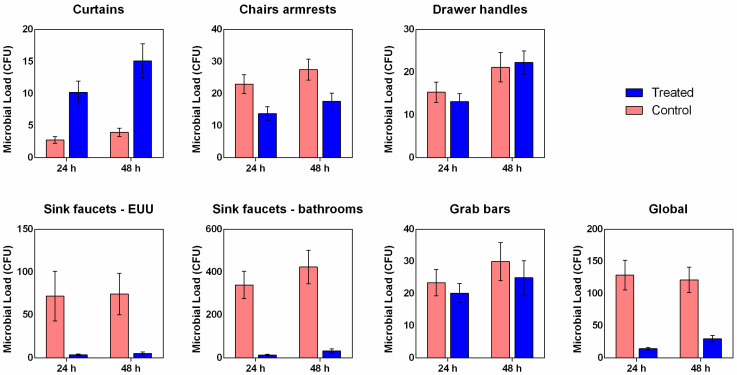
Mean microbial load recovered from hospital surfaces covered with copper-alloy adhesive tape and non-antimicrobial control tape. Data represent mean ± SEM of colony-forming unit (CFU).

The clinical study demonstrated that the application of the copper-alloy tape produced a marked reduction in global microbial load compared to controls, observed both 24 and 48 h of incubation (Figure 5). The sinks—both those located inside the EUU and those in the bathrooms—showed the highest mean microbial counts among all evaluated sites. This finding reflects the high contamination potential of these surfaces, associated with constant moisture and frequent handling by healthcare professionals and patients. Additionally, the most pronounced microbial reductions were observed on the sinks, particularly those in the bathrooms, where the treated group showed mean values of 13.05 ± 3.60 CFU at 24 h and 32.21 ± 9.51 CFU at 48 h, compared to the control group, which presented 339.50 ± 64.54 CFU and 423.37 ± 78.25 CFU, respectively (Figure 5 and Table S1).

On surfaces such as chair armrests, grab bars, and drawer handles, the differences between the groups control and copper-alloy tape were smaller (Figure 5 and Table S1), which may be related to the lower rate of microbial deposition or reduced microbial transfer due to less frequent contact. Interestingly, the counts for the curtains appear to be unique. The treated curtains showed comparable counts to those of the treated chairs and handles, both 24 h (10.20 ± 1.76, 3.77 ± 2.18, and 13.15 ± 1.85 CFU, respectively) and 48 h (15.10 ± 2.67, 17.59 ± 2.58, and 22.26 ± 2.72 CFU, respectively) of incubation (Figure 5 and Table S1). This suggests that the residual contamination level on the copper-alloy tape-coated curtains was similar to that of the other coated surfaces. However, the counts on the control curtains show a much lower baseline value than those on the chairs and handles, also located in the EUU patient unit. This may be related to less direct and frequent touching or even to the difficulty in attaching airborne microorganisms. These findings were statistically confirmed by both the Mann–Whitney U and independent-samples *t*-tests (Tables S2 and S3), which demonstrated significant reductions (*p* < 0.05) in overall microbial load on treated surfaces after both 24 h and 48 h incubations, supporting the antimicrobial efficacy of the copper-alloy coating.

**Figure 6:**
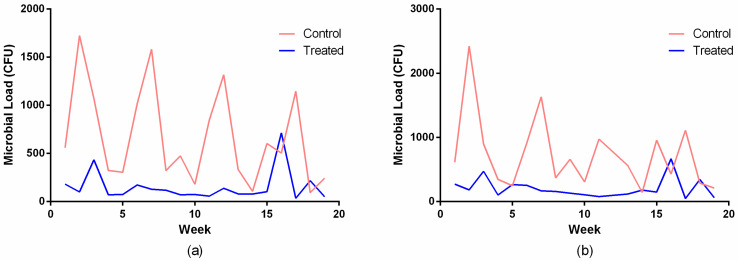
Temporal variation in global microbial load recovered from hospital surfaces covered with copper-alloy adhesive tape and non-antimicrobial control tape over a 19-week monitoring period, following incubation of 24 h (**a**) and 48 h (**b**).

Figure 6 illustrates the temporal variation in global microbial load over a 19-week period. The control group exhibited high week-to-week variability, reflecting real-world variations in surface use, environmental conditions, and cleaning conditions in the hospital unit. Although fluctuations were observed across the study period, a consistent pattern emerged in which the surfaces treated with the copper-alloy tape maintained markedly lower microbial loads than the untreated controls. This pattern was observed at both incubation times, confirming that the observed differences were independent of culture duration. Investigations of copper surfaces in hospital environments have reported significant reductions in contamination with multidrug-resistant strains, correlating with decreased incidence of healthcare-associated infections [7,22].

After 48 h of incubation, colonies were examined microscopically for yeast identification. As shown in Table 2, the number of yeast-positive samples was small compared to the total number of samples collected during the study. Yeasts were detected in at least one sample of all surfaces, except for the copper-alloy tape placed on the sink faucets in EUU. *Candida parapsilosis* was the most frequently identified species. This yeast has been frequently reported in mycological surveys in intensive care units, where it is associated with episodes of invasive candidemia. Its ability to persist in the hospital environment, primarily through biofilm formation, coupled with its increasing resistance to antifungal agents, has made *C. parapsilosis* a species of particular concern in hospital settings [23].

Although the assessment of yeasts in this study was qualitative, the data obtained will help to support future research, such as quantitative evaluations of the hospital environment, with the aim of better understanding the dissemination dynamics of the most frequently isolated species, as well as investigating their antimicrobial resistance profiles.

Overall, the results obtained in this study not only confirm the antimicrobial efficacy and sustained performance of the copper–polymer tape under both laboratory and hospital conditions, but also highlight its practical advantages for large-scale implementation. In contrast to solid copper, which is costly and mechanically rigid, adhesive copper–polymer composites can provide lightweight and flexible alternatives that are easily applied to existing surfaces without structural modifications [8]. The material can be quickly installed, replaced when worn, and maintained using conventional cleaning routines, representing a scalable and cost-effective strategy for long-term microbial control in healthcare environments. Recent findings with other copper-based composite coatings, such as the chitosan–nanoCu film described by Ayala-Peña et al. (2025) [24], further reinforce the versatility and antimicrobial potential of engineered copper materials, and our results extend this evidence by demonstrating sustained performance under real hospital conditions.

## 3. Materials and Methods

**Copper-based surface disinfectant.** The test material was a self-decontaminating adhesive tape (MS-Sticker^®^, MetalSkin Technologies, Toulouse, France), produced from an alloy containing a minimum of 92% copper (CAS 7440-50-8) combined with polymers. The tape consists of three layers: a protective rubber layer, a self-adhesive layer, and a functional upper layer containing the active ingredient.

**X-ray diffraction.** Copper-alloy tape was analyzed by XRD to determine copper crystalline structures. The measurements were conducted on a Rigaku (Tokyo, Japan) Ultima IV instrument with CuKα radiation. The diffraction patterns were recorded in the 20–80° range with a step size of 0.01° and a scan rate of 5°/min.

**Raman spectroscopy.** Copper-alloy tape was analyzed by Raman spectroscopy to corroborate the XRD data in determining the crystalline structures of copper and to assess their distribution along the sample. Raman spectra were acquired using an inVia™ Raman confocal microscope (Renishaw, Gloucestershire, UK) with a 532 nm laser (power of 10%), a 20× objective, and a grating of 1800 lines/mm. Single spectrum measurements covered the 100–900 cm^−1^ range, with a 10 s acquisition time and 5 accumulations. For Raman mapping, spectra were collected within the same spectral range, employing a 10 s acquisition time and 1 accumulation at each point. The mapped area, measuring x = 100 μm and y = 100 μm, comprised 225 data points (7 μm step size). Image generation involved mapping the intensity of the band related to Cu_2_O/CuO (633 cm^−1^) in each spectrum. All aspects of data handling, processing, and 3D color map reconstruction were performed using WiRE 5.6 software.

**Energy-dispersive X-ray spectroscopy coupled to Scanning Electron Microscopy (EDX-SEM).** The morphology of the copper-alloy tape surface was evaluated in a Scios 2 FIB-SEM Thermo Fisher Scientific microscope (Waltham, MA, USA). The composition and distribution of chemical elements were evaluated in a Bruker (Billerica, MA, USA) EDX system coupled to the FIB-SEM.

***In vitro* antimicrobial assays.** The antimicrobial activity of the copper-alloy tape was evaluated according to the ISO 22196:2011 standard (Measurement of antibacterial activity on plastics and other non-porous surfaces). Two types of samples were analyzed: (i) copper-containing adhesive tape (test sample) and (ii) adhesive tape without the antimicrobial component (control sample). Test microorganisms included *Escherichia coli* ATCC 8739, *Pseudomonas aeruginosa* ATCC 15442, *Salmonella enterica* subsp. enterica serovar Choleraesuis ATCC 10708, and *Staphylococcus aureus* ATCC 6538. For each assay, bacterial suspensions were prepared at approximately 10^9^ CFU/mL and inoculated onto the test and control surfaces. After an initial enumeration (time zero), samples were incubated for 24 h at 35 ± 1 °C under controlled conditions. Following incubation, viable bacterial counts were determined by serial dilution and plating. Results are expressed as colony-forming units per milliliter (CFU/mL), and the antibacterial activity was calculated as the logarithmic reduction in bacterial counts compared with the control, expressed both as log_10_ reduction and percentage reduction.

**Scanning Electron Microscopy analysis of bacteria**. For SEM analysis of bacteria on the copper-alloy tape, fragments of the tape (0.33 cm^2^) were sterilized by 70% ethanol and 30 min UV light exposure, glued to a glass cover slide, and positioned in a 12-well culture plate. A suspension of bacteria *Staphylococcus aureus* ATCC 25923 and *Salmonella typhimurium* ATCC 14028 (1 mL) at 10^6^ UFC/mL in Mueller-Hinton broth was added to each well, centrifugated at 3100× *g* for 5 min to deposit on the surface and incubated at 35 ± 2 °C for 30 min. After exposure time, the cells were washed with 0.1 M cacodylate buffer three times and fixed in Karnovsky’s fixative solution overnight at 5 °C. Cells were washed with cacodylate buffer, treated with osmium tetroxide (1%), and dehydrated using ethanol (25–100%) and hexamethyldisilazane (HMDS, 25, 50 and 100%). After air drying, cells were covered with ultra-thin coating of Au-Pd and examined by a Scios 2 FIB-SEM Thermo Fisher Scientific microscope (USA).

**Scanning Electron Microscopy analysis of human cells**. A human keratinocytes cell line (HaCaT) was acquired from Rio de Janeiro Cell Bank (BCRJ) and cultured in Dulbecco’s Modified Eagle’s Medium (DMEM) supplemented with 10% (*v*/*v*) FBS and 1% (*v*/*v*) antibiotic solution (5000 U/mL penicillin and streptomycin) at 37 °C and 5% CO_2_ in a humidified atmosphere. Fragments of copper-alloy tape (0.33 cm^2^) were sterilized by 70% ethanol and 30 min UV light exposure. The fragments were glued to a glass cover slide and positioned in a 12-well culture plate. Cells were seeded at 1 × 10^5^ cell/well, centrifugated at 140× *g* for 5 min to deposit on the surface, and incubated at 37 °C, 5% CO_2_ for 6 h. After exposure time, cells were washed with 0.1 M cacodylate buffer three times, fixed in Karnovsky’s fixative solution and post-fixed in 1% osmium tetroxide solution. Then, cells were dehydrated in increasing concentrations of acetone (30–100%), submitted to critical point drying (CPD 030, Balzers, Schaumburg, IL, USA) and covered with an ultra-thin coating of Au with the EM SCD 500 vacuum sputter coater (Leica Microsystems, Wetzlar, Germany). Images were acquired in a JSM-7001F Scanning Electron Microscope (Jeol, Tokyo, Japan).

**Randomized clinical assessment in hospital environment.** To evaluate the clinical efficacy of the copper-alloy tape, a quantitative randomized clinical study was conducted in the Emergency and Urgent Unit (EUU) of the University Hospital of Brasilia (HUB) in Brasilia City, Federal District, Brazil. The hospital unit is characterized by a semi-intensive care profile and the stay of patients in serious conditions and returns from the Intensive Care Unit. The study was conducted following evaluation and prior approval by the Research Ethics Committee of the Faculty of Medicine of University of Brasilia (approval no. 6634927) and in accordance with the guidelines of the National Health Council Resolutions n° 466/1996, n° 466/2012 and n° 510/2016.

Sampling sites were selected based on their high frequency of contact with healthcare staff and visitors, and included patient unit divider curtains (external surface), chair armrests (right and left), drawer handles of medication carts, sink faucets in the EUU, sink faucets in the bathrooms, and grab bars in the bathrooms (shower and toilet bowl). In total, 40 sampling sites were randomized using dedicated software to ensure unbiased allocation between the test and control groups. Twenty sites were assigned to the control group (adhesive tape without the antimicrobial component) and 20 sites to the test group (copper-containing adhesive tape). Both control and test tapes were standardized as circular disks (8 mm in diameter) and adhered to flat surfaces at randomized sites. After adhesion, all surfaces were cleaned with 70% ethanol following hospital hygiene standards.

In order to reflect real-world environmental conditions, the hospital’s standard hygiene and disinfection protocol was maintained throughout the study and performed daily. The procedure consisted of wiping surfaces with a white disposable multipurpose cloth moistened with Peroxy 4D, an intermediate-level disinfectant formulated with fifth-generation quaternary ammonium compounds and hydrogen peroxide. Terminal cleaning procedures, including thorough washing of the entire patient unit, were carried out at varying frequencies and locations following patient discharge.

Microbiological sampling was performed using RODAC plates containing HiCrome UTI agar. For each collection, the agar surface was directly pressed against the exposed surface of the tapes (test and control). Samples were transported under aseptic conditions to the laboratory, where they were incubated at 35 ± 2 °C for 24 and 48 h. Colony-forming units (CFU) were then quantified and characterized according to their morphology. Microbiological samples were collected twice weekly for 19 consecutive weeks.

**Yeast Isolation and Identification.** Since UTI Agar does not allow yeast differentiation, the plates were screened using microscopic evaluation. Using a bacteriological loop, all colonies were collected from the agar surface and resuspended in a microtube containing 500 microliters of 0.85% NaCl solution. The microtubes were vigorously shaken on a vortex mixer, and an aliquot of the suspension was evaluated under an optical microscope at 400× magnification for identification of suggestive yeast-like structures. After evaluation, a loop of the samples with suggestive yeast presence was plated on chromogenic agar (CHROMAgar Candida), and colonies were isolated from successive subcultures on the same medium. The plates were incubated at 37 °C and evaluated after 24 h and 48 h of incubation. After purification, colonies grown at 37 °C for 24 h were identified by mass spectrometry (VITEK-MS, Marcy-l’Étoile, France, Biomérieux). Cells were prepared according to the manufacturer’s instructions. A loopful of individual colonies was applied to a metal slide and lysed with 0.5 microliters of 28.9% formic acid. After drying at room temperature, 1 microliter of MS-CHCA matrix was added. After drying again, the slide was evaluated on a VITEK-MS instrument, and the results were evaluated using the clinical knowledge base. An *Escherichia coli* strain (ATCC 8739) was used as the standard. All reagents were purchased from Biomérieux.

**Statistics.** Data normality was assessed using the Shapiro–Wilk test. Depending on data distribution, comparisons between treated and control groups were performed using either the independent-samples Student’s *t*-test or the nonparametric Mann–Whitney *U* test. Statistical significance was considered at *p* < 0.05 for all comparisons. The global variable, representing the mean microbial load across all surfaces, was also included in the analysis. Data were processed using the GraphPad Prism version 8.4.3 (GraphPad Software, San Diego, CA, USA) and results are presented as mean ± standard deviation or standard error of the mean.

## 4. Conclusions

In conclusion, the copper-alloy tape for surface disinfectant applications demonstrated significant antimicrobial efficacy against a wide spectrum of clinically relevant microorganisms, both in controlled *in vitro* assays and real-world hospital surface. Structural characterization techniques—including XRD, Raman spectroscopy, SEM, and EDX—validated the integrity and heterogeneous distribution of copper within the polymeric matrix, consistent with enhanced biocidal performance.

In real-world hospital conditions, the copper-alloy polymeric tape consistently maintained low microbial counts over a 19-week monitoring period, even under variable environmental and cleaning conditions. The significant reductions in bacterial load observed across multiple surface types, particularly on high-risk areas such as sinks and faucets, confirm its robust and sustained antimicrobial performance. The qualitative yeast analysis also indicated reduced fungal persistence on treated surfaces, supporting its broad-spectrum potential.

Taken together, these findings highlight the copper-alloy tape as a durable, passive, and effective surface-disinfection technology for infection control in healthcare environments. Future studies should explore its long-term stability under different humidity and abrasion conditions and its impact on overall hospital infection rates.

## Figures and Tables

**Table 1:** Antibacterial activity of the copper-alloy tape according to ISO 22196:2011.

Bacterial Strain	Control (log10 CFU/mL)	Test (log10 CFU/mL)
*Escherichia coli* ATCC 8739	5.33 ± 0.61	<1.00
*Pseudomonas aeruginosa* ATCC 15442	5.22 ± 0.54	<1.00
*Salmonella enterica* subsp. enterica serovar Choleraesuis ATCC 10708	3.59 ± 0.53	<1.00
*Staphylococcus aureus* ATCC 6538	1.66 ± 0.72	<1.00

Values represent mean ± standard deviation (SD) of five replicates, after 24 h exposure.

**Table 2:** Yeast species isolated from colonies grown after 48 h of incubation from environmental samples collected on hospital surfaces in the Emergency and Urgency Unit (EUU) of the Hospital.

Variable	Group	Positive Samples	Yeast Species
Patient unit divider curtains	Control	2/75	*Candida parapsilosis* (2/2).
	Test	6/75	*Candida parapsilosis* (3/6), *Trichosporon ovoides* (1/6), *Trichosporon asahii* (1/6), *Rhodotorula mucilaginosa* (1/6).
Chair armrests	Control	6/75	*Candida parapsilosis* (5/6), not identified (1/6).
	Test	4/75	*Candida parapsilosis* (2/4), *Candida orthopsilosis* (1/4), *Trichosporon asahii* (1/4).
Drawer handles of medication carts	Control	3/90	*Candida parapsilosis* (3/3).
	Test	5/90	*Candida parapsilosis* (5/5).
Sink faucets in the EUU	Control	2/15	*Candida parapsilosis* (1/2), *Candida guilliermondii* (1/2).
	Test	0/15	-
Sink faucets in the bathrooms	Control	4/15	*Candida parapsilosis* (2/4), *Candida glabrata* (1/4), *Candida parapsilosis + Candida albicans* (1/4).
	Test	1/15	*Candida parapsilosis* (1/4).
Grab bars in the bathrooms (shower and toilet bowl)	Control	1/30	*Candida parapsilosis* (1/1).
	Test	4/30	*Candida parapsilosis* (2/4), *Candida parapsilosis + Candida albicans* (1/4), *Candida albicans* (1/4).

Data represent the number of positive samples over the total samples collected at each site. Species identification was based on colony morphology and biochemical characterization. The control group refers to surfaces covered with a non-antimicrobial adhesive tape, whereas the test group refers to surfaces covered with the copper-alloy tape.

## Data Availability

The raw data supporting the conclusions of this article will be made available by the authors on request.

## Supplementary Materials

The following supporting information can be downloaded at: https://www.mdpi.com/article/10.3390/antibiotics14121262/s1.
