# Supplementary material for: Antimicrobial Activity and Characterization of a Validated Copper-Complexed Polymer Tape for Surface Disinfectant Applications
Source: Antibiotics (Basel). 2025 Dec 14;14(12):1262. doi: 10.3390/antibiotics14121262 (PMC12729683; doi:10.3390/antibiotics14121262)
Supplement: Supplementary file 1 [file antibiotics-14-01262-s001.zip › antibiotics-3948389-supplementary.pdf]

Supplementary material

Antimicrobial Activity and Characterization of a Validated Copper-Complexed Polymer Tape for Surface Disinfectant Applications

Andreanne G. Vasconcelos [1,2,3,4,*], William D. Amorim [3], Bruno S. Sá [2], Luan B. V. Costa [2], Gustavo S. de Araujo [5], Helder Andrey R. Gomes [3,6], Jorge Antônio Chamon Júnior [6], Amabel F. Correia [6], Íris Cabral [6], Thales R. Machado [7], Dayse Maria C. de Mendonça [8], Ingrid Gracielle M. da Silva [9], Joaquim L. Júnior [10], Elivaldo R. de Santana [11], Yvonne Mascarenhas [12], Sônia N. Báo [9], Valtencir Zucolotto [7], Peter Eaton [4], Ciro M. Gomes [13] and José Roberto de S. A. Leite [1,2,*]

[1] People&Science Pesquisa Desenvolvimento e Inovação Ltda, Centro de Apoio ao Desenvolvimento Tecnológico (CDT), University of Brasília (UnB), Brasília 70910-900, DF, Brazil
[2] Research Center in Applied Morphology and Immunology (NuPMIA), Faculty of Medicine, University of Brasília (UnB), Brasília 70910-900, DF, Brazil
[3] Centro Universitário do Distrito Federal (UDF), Brasília 70390-045, DF, Brazil
[4] The Bridge, University of Lincoln, Joseph Ruston Building, Lincoln LN6 7EL, UK; peaton@lincoln.ac.uk
[5] Programa de Pós-Graduação em Patologia Molecular, Faculty of Medicine, University of Brasília (UnB), Brasília 70910-900, DF, Brazil
[6] Laboratório Central de Saúde Pública (LACEN-DF), Brasília 70830-010, DF, Brazil
[7] Nanomedicine and Nanotoxicology Group, São Carlos Institute of Physics, University of São Paulo (USP), São Carlos 13566-590, SP, Brazil; zuco@ifsc.usp.br (V.Z.)
[8] Gerência de Atenção à Saúde, Unidade de Clínica Médica, Hospital Universitário de Brasília (HUB), University of Brasília (UnB), Brasília 72830-200, DF, Brazil
[9] Laboratório de Microscopia e Microanálise, Instituto de Ciências Biológicas, University of Brasília (UnB), Brasília 70910-900, DF, Brazil; snbao@unb.br (S.N.B.)
[10] Programa de Epidemiologia e Vigilância em Saúde (PEPIVS), Fundação Oswaldo Cruz (Fiocruz), Brasília 70910-900, DF, Brazil
[11] Laboratório de Simulação da Faculdade de Medicina, University of Brasília (UnB), Brasília 70910-900, DF, Brazil
[12] São Carlos Institute of Physics, University of São Paulo (USP), São Carlos 13566-590, SP, Brazil; yvonne@ifsc.usp.br
[13] Unidade de Dermatologia, Hospital Universitário de Brasília (HUB), University of Brasília (UnB), Brasília 72830-200, DF, Brazil; cirogomes@unb.br
* Correspondence: agomesvasconcelos@lincoln.ac.uk (A.G.V.); jrleite@unb.br (J.R.d.S.A.L.)

Normality was assessed using the Shapiro–Wilk test for each data group, given the sample size of fewer than thirty observations. After removing outliers identified by the Interquartile Range (IQR) method, statistical analyses were performed to evaluate differences between surfaces covered with the copper-alloy adhesive tape (test group) and those covered with the non-antimicrobial control tape, considering incubation times of 24 h and 48 h (Table S1). When data followed a non-normal distribution, the nonparametric Mann–Whitney *U* test was used (Table S2); when the distribution was normal, the independent-samples Student's *t*-test was applied (Table S3). Statistically significant differences ($p < 0.05$) between treated and control surfaces were observed for several surface types, particularly for curtains, sink faucets inside the Emergency and Urgent Unit (EUU), bathroom sink faucets, and in the

global analysis (considering all sampling sites). Comparisons between 24 h and 48 h incubation times showed that, overall, there was no substantial increase in microbial load on treated surfaces, whereas the control group exhibited higher counts or greater variability between incubation times.

Table S1. Descriptive statistics of colony-forming unit (CFU) count obtained from environmental surface samples collected in the Emergency and Urgency Unit (EUU) of the Hospital for 19 consecutive weeks. A total of 40 sampling sites were randomized, with 20 assigned to the control (non-antimicrobial tape) and 20 to the test (copper-alloy tape) groups.

Surface	Group	Mean	Standard deviation	Standard error of the mean
Patient unit divider curtains	Control 24 h	2.77	2.22	0.52
	Control 48 h	3.95	2.60	0.65
	Copper-alloy tape 24 h	10.20	7.49	1.76
	Copper-alloy tape 48 h	15.10	11.01	2.67
Chairs armrests	Control 24 h	22.97	12.64	2.90
	Control 48 h	27.51	13.05	3.26
	Copper-alloy tape 24 h	13.77	9.24	2.18
	Copper-alloy tape 48 h	17.59	9.99	2.58
Drawer handles of medication carts	Control 24 h	15.32	10.10	2.38
	Control 48 h	21.15	13.77	3.44
	Copper-alloy tape 24 h	13.15	7.63	1.85
	Copper-alloy tape 48 h	22.26	10.91	2.72
Sink faucets in the EUU	Control 24 h	71.93	115.90	28.97
	Control 48 h	74.33	93.76	24.21
	Copper-alloy tape 24 h	3.56	4.33	1.08

	Copper-alloy tape 48 h	5.33	6.20	1.60
Sink faucets in the bathrooms	Control 24 h	339.50	273.83	64.54
	Control 48 h	423.37	313.01	78.25
	Copper-alloy tape 24 h	13.05	14.85	3.60
	Copper-alloy tape 48 h	32.21	35.59	9.51
Grab bars in the bathrooms (shower and toilet bowl)	Control 24 h	23.36	17.59	4.14
	Control 48 h	29.91	24.40	5.91
	Copper-alloy tape 24 h	20.08	12.58	2.96
	Copper-alloy tape 48 h	24.87	21.15	5.28
Global	Control 24 h	128.60	100.54	23.06
	Control 48 h	121.11	81.59	19.79
	Copper-alloy tape 24 h	14.27	6.78	1.69
	Copper-alloy tape 48 h	29.75	20.02	4.85

Table S2. Nonparametric comparison by Mann_whitney U test of colony-forming unit (CFU) count obtained from environmental surface samples collected in the Emergency and Urgency Unit (EUU) of the Hospital with non-antimicrobial tape (control) and copper-alloy tape (test).

Surface	Comparison	Mann–Whitney U	p-value
Patient unit divider curtains	Control 24 h *vs*. Copper-alloy tape 24 h	36.000	$p < 0.05$
	Control 48 h *vs*. Copper-alloy tape 48 h	28.500	$p < 0.05$
	Copper-alloy tape 24 h *vs*. Copper-alloy tape 48 h	108.500	$p > 0.05$
	Control 24 h *vs*. Control 48 h	85.500	$p < 0.05$
Drawer handles of medication carts	Control 24 h *vs*. Copper-alloy tape 24 h	161.000	$p > 0.05$
	Control 48 h *vs*. Copper-alloy tape 48 h	104.000	$p > 0.05$
	Control 24 h *vs*. Control 48 h	103.000	$p > 0.05$
Sink faucets in the EUU	Control 24 h *vs* Copper-alloy tape 24 h	226.000	$p < 0.05$
	Control 48 h *vs*. Copper-alloy tape 48 h	198.000	$p < 0.05$
	Copper-alloy tape 24 h *vs*. Copper-alloy tape 48 h	105.000	$p > 0.05$
	Control 24 h *vs*. Control 48 h	106.000	$p > 0.05$
Sink faucets in the bathrooms	Control 24 h *vs*. Copper-alloy tape 24 h	302.000	$p < 0.05$
	Control 48 h *vs*. Copper-alloy tape 48 h	217.000	$p < 0.05$
	Copper-alloy tape 24 h *vs*. Copper-alloy tape 48 h	70.000	$p > 0.05$
	Control 24 h *vs*. Control 48 h	124.500	$p > 0.05$
Global	Control 24 h *vs*. Copper-alloy tape 24 h	291.000	$p < 0.05$

	Control 48 h *vs*. Copper-alloy tape 48 h	264.000	$p < 0.05$
	Copper-alloy tape 24 h *vs*. Copper-alloy tape 48 h	59.000	$p < 0.05$
	Control 24 h *vs*. Control 48 h	159.000	$p > 0.05$

Statistically significant results are shown as $p < 0.05$.

Table S3. Independent Student's *t*-test of colony-forming unit (CFU) count obtained from environmental surface samples collected in the Emergency and Urgency Unit (EUU) of the Hospital with non-antimicrobial tape (control) and copper-alloy tape (test).

Variables	Groups	t-Statistic	p-value
Grab bars in the bathrooms (shower and toilet bowl)	Control 24 h *vs*. Copper-alloy tape 24 h	0.642	$p > 0.05$
	Control 48 h *vs*. Copper-alloy tape 48 h	0.631	$p > 0.05$
	Copper-alloy tape 24 h *vs*. Copper-alloy tape 48 h	-0.813	$p < 0.05$
	Control 24 h *vs*. Control 48 h	-0.914	$p < 0.05$
Chairs armrests	Copper-alloy tape 24 h *vs*. Copper-alloy tape 48 h	2.514	$p < 0.05$
	Control 48 h *vs*. Copper-alloy tape 48 h	2.362	$p < 0.05$
	Copper-alloy tape 24 h *vs*. Copper-alloy tape 48 h	-2.791	$p < 0.05$
	Control 24 h *vs*. Control 48 h	-1.041	$p > 0.05$
Drawer handles of medication carts	Copper-alloy tape 24 h *vs*. Copper-alloy tape 48 h	-2.791	$p < 0.05$

Statistically significant results are shown as $p < 0.05$.
